# Effect of Theta Transcranial Alternating Current Stimulation and Phase-Locked Transcranial Pulsed Current Stimulation on Learning and Cognitive Control

**DOI:** 10.3389/fnins.2019.01181

**Published:** 2019-11-14

**Authors:** Farrokh Mansouri, Alaa Shanbour, Frank Mazza, Peter Fettes, José Zariffa, Jonathan Downar

**Affiliations:** ^1^Institute of Biomaterials and Biomedical Engineering, University of Toronto, Toronto, ON, Canada; ^2^Department of Psychiatry, Central Michigan University, Mount Pleasant, MI, United States; ^3^Institute of Medical Science, University of Toronto, Toronto, ON, Canada; ^4^Department of Physiology, University of Toronto, Toronto, ON, Canada; ^5^KITE, Toronto Rehab, University Health Network, Toronto, ON, Canada; ^6^Rehabilitation Sciences Institute, University of Toronto, Toronto, ON, Canada; ^7^Edward S. Rogers Sr. Department of Electrical and Computer Engineering, University of Toronto, Toronto, ON, Canada; ^8^Department of Psychiatry, University of Toronto, Toronto, ON, Canada; ^9^Centre for Mental Health, University Health Network, Toronto, ON, Canada; ^10^Krembil Research Institute, University Health Network, Toronto, ON, Canada

**Keywords:** brain stimulation, transcranial alternating current stimulation, transcranial pulsed current stimulation, transcranial electrical stimulation, phase-locked brain stimulation, closed-loop brain stimulation, salience network

## Abstract

Non-invasive brain stimulation (NIBS) is emerging as a robust treatment alternative for major depressive disorder, with a potential for achieving higher remission rates by providing targeted stimulation to underlying brain networks, such as the salience network (SN). Growing evidence suggests that these therapeutic effects are dependent on the frequency and phase synchrony between SN oscillations and stimulation as well as the task-specific state of the SN during stimulation. However, the development of phase-synchronized non-invasive stimulation has proved challenging until recently. Here, we use a phase-locked pulsed brain stimulation approach to study the effects of two NIBS methods: transcranial alternating current stimulation (tACS) versus phase-locked transcranial pulsed current stimulation (tPCS), on the SN during an SN activating task. 20 healthy volunteers participated in the study. Each volunteer partook in four sessions, receiving one stimulation type at random (theta-tACS, peak tPCS, trough tPCS or sham) while undergoing a learning game, followed by an unstimulated test based on learned material. Each session lasted approximately 1.5 h, with an interval of at least 2 days to allow for washout and to avoid cross-over effects. Our results showed no statistically significant effect of stimulation on the event related potential (ERP) recordings, resting electroencephalogram (EEG), and the performance of the volunteers. While stimulation effects were not apparent in this study, the nominal performance of the phase-locking algorithm offers a technical foundation for further research in determining effective stimulation paradigms and conditions. Specifically, future work should investigate stronger stimulation and true task-specific stimulation of SN nodes responsible for the task as well as their recording. If refined, NIBS could offer an effective, homebased treatment option.

## Introduction

Non-invasive brain stimulation (NIBS) is an emerging alternative when conventional treatment approaches for Major Depressive Disorder (MDD) fail (Fregni and Pascual-Leone, 2007; Daskalakis et al., 2008; Ferrucci et al., 2009; Dayan et al., 2013). It is estimated that one in three MDD patients suffers from Treatment Resistant Depression (TRD), with failure to respond to at least two courses of antidepressant treatment (Nemeroff, 2007). NIBS technologies such as Food and Drug Administration (FDA)-approved repetitive transcranial magnetic stimulation (rTMS) and transcranial direct current stimulation (tDCS) have shown efficacy in helping with TRD in a large number of clinical trials, achieving 15–32% (O’Reardon et al., 2007; Berlim et al., 2014; Gaynes et al., 2014) and 7–43% (Berlim et al., 2013; Shiozawa et al., 2014) remission rates, respectively. There is a potential for achieving higher remission rates by providing a more targeted stimulation to the brain networks involved in MDD. Growing evidence (reviewed below) suggests that this can be achieved by first applying the stimulation at the same oscillatory profile as the underlying brain oscillation, and second by applying the stimulation during the time when the brain network of interest is in the most suitable state to receive the stimulation.

In contrast to tDCS, transcranial alternating current stimulation (tACS) and transcranial pulsed current stimulation (tPCS) use oscillatory waveforms with sinusoidal and square pulses that better match the underlying natural physiological brain activity. In a recent study, it has been shown that tACS can have an effect in spike timing of single neurons elucidating the mechanism of action for these types of NIBS and opening the opportunity for future research into the effects of timing of the stimulation waveform with respect to the underlying brain activity (Krause et al., 2019).

Multiple studies have shown the importance of achieving synchrony between the target brain oscillation and the stimulation frequencies and phase in order to increase the effect of the stimulation. A recent study has shown that adjusting rTMS pulse frequency to individual gamma oscillation resulted in a significant mood elevation compared to unadjusted rTMS stimulation at slightly higher or lower frequencies than their individual gamma oscillation (Chung et al., 2018). In another example, TMS phase-specific modulation of motor evoked potentials has been shown by applying pulses at the peak or trough of the μ-rhythm of the motor cortex (Zrenner et al., 2017). Similarly, the brain stimulation modalities that use energies below the threshold for induction of an action potential (e.g., transcranial electrical stimulation), are more effective when delivered at similar oscillation frequencies as the underlying brain target (Fröhlich and McCormick, 2010; Reato et al., 2013). For example, tACS has been shown effective to modulate alpha power only when delivered at alpha frequency (Zaehle et al., 2010; Vossen et al., 2015). Further, tACS delivered at a specific phase with respect to the underlying brain oscillation modulated the intensity of the tremor when applied to the motor cortex (Brittain et al., 2013) and changed the hearing thresholds when applied to the auditory cortex (Riecke et al., 2015; Wilsch et al., 2018).

The effects of brain stimulation have also consistently been shown to depend on the brain state at the time of stimulation (Silvanto et al., 2008). Current models of brain function posit that brain regions operate as integrated networks bound by coherent activity, and task-specific activation of these networks is seen across various brain states (Seager et al., 2002; Park and Friston, 2013; Pessoa, 2014). The state of the brain during the stimulation can change the outcome of the intervention; an elementary example would be the observation that the active motor threshold is substantially lower than the resting motor threshold for stimulation of the primary motor cortex (Hallett, 2007).

A useful stimulation target is the Salience Network (SN), which is activated when there is a transition between a cognitive task and sensory information (Downar et al., 2000, 2001). SN dysfunction is associated with a wide range of neuropsychiatric disorders, including MDD, post-traumatic stress disorder (PTSD), and schizophrenia (Peters et al., 2016). Targeting the SN has been proven successful when using rTMS for treating MDD (Peters et al., 2016). Considering the importance of brain state during brain stimulation, applying the stimulation during an SN-activating task may potentially improve the effect of the stimulation.

Providing a more targeted brain stimulation may help discover more effective treatments for MDD patients. A closed-loop system enabling phase-locked stimulation could potentially allow more precise control of the stimulation frequency and phase. Such system could achieve a more consistent treatment effect overall, given the findings pointing at the importance of brain state during the stimulation and the synchrony of the stimulation with the underlying brain oscillation. Here, we conducted an experiment to study the effect of two NIBS methods: tACS and transcranial phase-locked pulsed current stimulation (tPCS), on the SN during an SN activating task. The effects of these types of stimulation have previously been shown to be: spectral power density changes in specific frequency bands during rest EEG (Zaehle et al., 2010; Helfrich et al., 2014; Vossen et al., 2015), changes in task-specific activation of the brain (Meinzer et al., 2012; Jaušovec and Jaušovec, 2014; Cabral-Calderin et al., 2016), and behavioral or task-specific performance changes (Santarnecchi et al., 2013; Voss et al., 2014). We, therefore, hypothesized that by providing stimulation in synchrony with the underlying brain activity during an SN activating task, we would achieve more effective stimulation of the SN, as reflected by increases in these previously established electrophysiological and behavioral effects. We further hypothesized that tACS would strengthen the SN activity resulting in faster reaction time and better performance during the task, while producing an increase in resting theta power and ERP.

In this work we attempted to engage the SN by using tACS at theta frequency or phase-locked tPCS synchronized to the frontal theta when the volunteers were engaged in a SN activating task. Successful implementation of tACS in a closed-loop system with electroencephalography (EEG) recording is currently an unsolved problem, because the artifact generated by the stimulation obscures the recording (Neuling et al., 2017; Noury and Siegel, 2017). To study the phase- and frequency-locked brain stimulation method, we previously have developed a phase-locked tPCS brain stimulation technique that can extract phase and frequency of the EEG signal and deliver the stimulation pulses at specific phase and frequency of the EEG signal in real-time (Mansouri et al., 2018). Here we applied this technique to phase-lock tPCS to the activity of the SN recorded through theta EEG. The electrical pulses were delivered either at peak or trough of the recording, with the expected result of generating opposite effects – as was previously shown when using rTMS over motor cortex (Zrenner et al., 2017), electrical stimulation of hippocampal brain slices (Hyman et al., 2003), and electrical stimulation of the somatosensory cortex in monkeys (Zanos et al., 2018). We hypothesized that trough tPCS would strengthen the SN activity, while peak tPCS would have the opposite effect, and that these effects would be exhibited through an increase or decrease of theta power and ERP recording, alongside changes in behavioral performance during the task.

## Materials and Methods

### Participants and Visits

Twenty healthy adult (older than 18) volunteers free of neurological or psychiatric illnesses participated in the study. All participants gave written informed consent, and the study was approved by the Research Ethics Board of the University Health Network. For each of the four visits, volunteers received one of the stimulation types at random (theta-tACS, peak tPCS, trough tPCS or sham). The order of the visits was assigned at random using a random generator with the participants not informed of the order of the visits; only the experimenter knew the type of stimulation at each visit. The visits were scheduled at least 2 days apart to allow for washout and to avoid cross-over effects. Each session took approximately 1 h and a half.

### Study Visit and Computer Task

**FIGURE 1:**
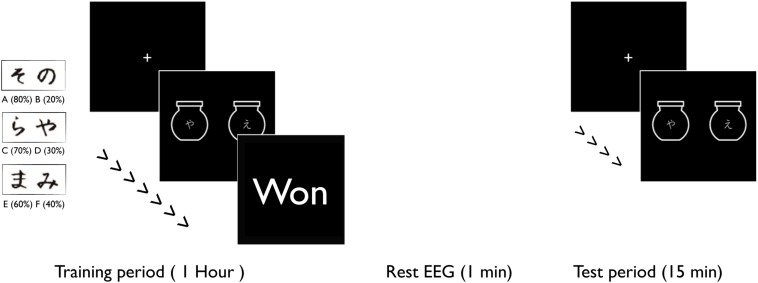
Volunteers were presented with six pairs of Japanese characters. In each pair, one character had a higher probability of “winning”. During the training period, the volunteers were presented with these pairs to learn their probabilities through reinforcement learning. The training task was continued for 1 h. The volunteers completed 1 min of rest EEG recording after the training period. During the test period volunteers, were presented with two Japanese characters but not necessarily paired in the same way, to create conflicting scenarios (win/win or lose/lose).

During each visit, volunteers sat in front of a computer and performed a decision-making task described by Frank et al. (2004, 2005). Each visit consisted of a learning and a test period (Figure 1). During the learning period, the volunteers were presented with three pairs of randomly assigned Japanese characters (AB, CD, and EF) that were not easily verbalized. In each pair, one of the characters was more likely to win a reward (A:80%-B:20%, C:70%-D:30% and E:60%-F:40%). For each trial, the volunteers were presented with one pair (each character was displayed on an image of a jar) and within 1 sec had to select a character by pressing the left or the right arrow keys on a keyboard. Next, the volunteers were presented with a feedback to tell them whether they “won”, “lost”, or were too late to respond. This game continued for 1 h, with 1 min breaks every 4 min.

After completing the learning game, volunteers took a short test with same pairs as the learning game (AB, CD, and EF) and also pairs that they had not previously seen (AC, AE, CE, BD, BE, and DE). During the test, no feedback was presented, to avoid further learning effects.

The same task was performed during each of the four sessions. For each session, six new randomly selected Japanese characters were used. At each session, during the learning task, the volunteers received one of the 4 stimulation types. No stimulation was delivered during the test task.

### Phase-Locked Transcranial Pulsed Current Stimulation

**FIGURE 2:**
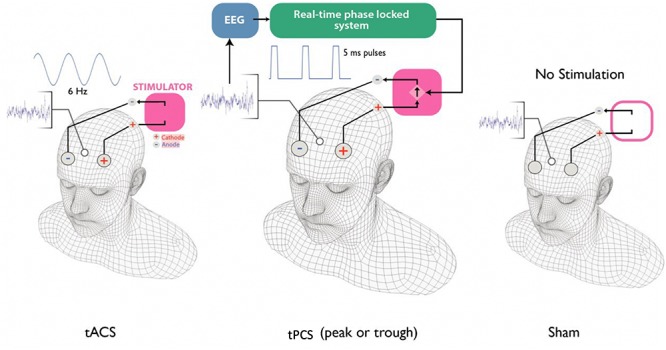
Four different stimulation types were used in this study: tACS, tPCS peak, tPCS trough and sham. During tACS stimulation, a 6 Hz sinusoidal stimulation was applied over F3 and F4. During the tPCS at peak or trough, 5 ms pulses were delivered at peak or trough of the theta oscillation recorded from Fz. During the sham session, no stimulation was delivered.

A similar method to Mansouri et al. (2018) was used to provide square wave 5 ms pulses of 2 mA amplitude at either peak (90 degree phase) or trough (270 degree phase) of the theta oscillation (4–8 Hz) recorded from the midfrontal part of the scalp. First, the stimulation electrodes (2 cm × 2 cm) were placed on the scalp at F3 (anode) and F4 (cathode); Ten20 conductive gel (Weaver and Company, Aurora, CO., United States) was applied to reduce the impedances of the electrodes to below 5 kΩ. Next, a 16-channel passive-electrode EEG cap (EasyCap GmbH, Germany) was worn by the volunteers on top of the stimulation electrodes and HiCL Abrasive EEG Gel (EasyCap GmbH, Germany) was applied to Fz (recording electrode), Pz (Reference electrode) and right tragus (Ground) to reduce the impedance of each of the recording electrodes to below 5 kΩ (Figure 2).

As previously described in Mansouri et al. (2017), the recorded EEG from Fz using V-AMP16 (Brain Products GmbH, Munich, Germany) was analyzed in MATLAB (MATLAB version 7.9.0) using a Fast Fourier Transform-based forecasting method (Mansouri et al., 2017) to provide the timing of the pulses and this timing was communicated to the ANT Neuro stimulator through an Arduino interface. The stimulation was applied for 1 h during the learning task; no stimulation was delivered during the test task.

### Transcranial Alternating Current Stimulation (tACS) Procedure

First, the stimulation electrodes (2 cm × 2 cm) were placed on the scalp at F3 (anode) and F4 (cathode); Ten20 conductive gel (Weaver and Company, Aurora, CO., United States) was applied to reduce the impedances of the electrodes to below 5 kΩ. Then, the stimulation was set to provide a 6 Hz sinusoidal current stimulation with amplitude of 2 mA peak to peak and zero direct current. The stimulation was initiated with a 30-s ramp up from zero to 2 mA amplitude. As in the tpES case, the stimulation was applied for 1 h during the learning task and no stimulation was delivered during the test task.

### Sham Stimulation Procedure

The stimulation electrodes were placed in a similar way to the pulsed stimulation and tACS stimulation. An initial tACS for 1 min with 30 s ramp up to 2 mA and 30 s ramp down stimulation was applied to help with the blinding of the participants. No further stimulation was applied during this visit. The participants completed both the learning and the test task.

### Resting-State EEG and ERP Recording

The same EEG setup was used for rest EEG and ERP recordings. Pz was used as the reference and right tragus as the ground electrodes. Rest EEG was recorded before the test task for 1 min as the volunteers sat in front of the computer and the recording continued to capture the event-related potential (ERP) during the test task. Trigger signals were provided to the amplifier to capture the events during the game.

### Resting-State EEG and ERP Analysis

All the analysis was done in MATLAB (MATLAB R2016b) and EEGLAB EEG analysis toolbox (Delorme and Makeig, 2004) was used for specific analysis. First, the channels were manually inspected and bad channels (large noise or poor connection) were identified and removed from the analysis. An average reference transformation was applied to the data to minimize the effect of reference site. Next a zero-phase 1 Hz high-pass FIR filter was used to remove the baseline drift. We applied a threshold to identify and remove large movement and eyeblink artifacts. Further, a zero-phase shift 1–50 Hz FIR bandpass filter was applied to the data. Response-locked ERP measures were extracted and a zero-phase shift 1–14 Hz bandpass IIR filter was applied to avoid bifurcation (Frank et al., 2005). Welch’s power spectral density estimate with a Hanning window was used to generate the spectral density of the rest EEG.

### Analysis

#### Statistical Methods and Analysis

Considering the design of the study, a repeated-measure method to investigate the variability within the factors (subjects) is suitable for testing the effect of the stimulation. Previous publications, that employed the same task with a larger group of participants, used a parametric test for their statistical evaluations (Frank et al., 2004, 2005). We performed a Shapiro–Wilk test, which refuted that our data is from a normally distributed population. Thus, instead of the parametric repeated-measure analysis of variance (ANOVA), the non-parametric repeated-measure Friedman’s test was used to assess the effect of the stimulation type over the outcome measures. Significance *p*-value level was set at 0.05 for rejecting the null hypothesis (no effect of stimulation). Considering the 15 statistical tests performed on the data, the significance *p*-value level with multiple comparison Bonferroni correction was adjusted to 0.003.

Phase Locking Value (PLV) was used to evaluate the performance of the phase-locked tPCS. PLV is a value between 0 and 1; higher PLV shows better phase locking.

#### Sample Size Justification

A power analysis for repeated-measure ANOVA test within factors was conducted using G^∗^Power (Faul et al., 2007) assuming an intermediate effect size (*f* = 0.5) based on previously published tDCS studies (Minarik et al., 2016; Aleman et al., 2018), 95% power and alpha error probability of 5%. This analysis suggested the total sample size of at least 20 participants. We assumed there were no carryover effects between the four sessions.

## Results

### Recruitment

Twenty volunteers (10/10 male/female; mean age 31.7 ± SD 8.6 years, range 23–53) were recruited to complete four visits. There were no complications after the stimulation sessions; minor tingling was reported during tACS stimulation. Pulsed stimulation was felt only during the beginning of the session as a tapping sensation on the head; the volunteers gradually acclimatized to these effects and reported no sensation of the stimulation afterward.

### Closed-Loop Brain Stimulation

Both peak and trough tPCS were applied successfully in terms of phase-locking performance. A minimum of 0.31 PLV and maximum of 0.70 PLV and average of 0.50 ± 0.13 was achieved for peak stimulation. A minimum of 0.32 PLV and maximum of 0.80 PLV and average of 0.53 ± 0.14 was achieved for trough stimulation. The average error in the peak stimulation was 2.5^o^ ± 16^o^ and for trough was 5.2^o^ ± 17^o^.

### Task-Specific Findings

All participants learned the probabilistic reward associations of the task successfully and were able to score more than 65% on AB cases, 60% on CD cases and 50% on EF cases during the learning task in all four visits. However, when comparing learning performance across the four stimulation conditions, no significant effect was apparent: there was no effect of stimulation type on decision of the players to choose A over B (Friedman’s test χ^2^_F_(3) = 1.49, *p* = 0.68), C over D (Friedman’s test χ^2^_F_ (3) = 4.02, *p* = 0.26), and E over F (Friedman’s test χ^2^_F_(3) = 5.84, *p* = 0.12).

Next, we examined at the probability of selecting A over all the other characters and probability of avoiding B over all the characters. “Positive learners”, as described by Frank et al., 2004 have stronger tendency to select A, while “negative learners” have stronger tendency to avoid B (Frank et al., 2004). We did not find any statistical effect of stimulation on probability of choosing A (Friedman’s test χ^2^_F_(3) = 1.76, *p* = 0.62), or avoiding B (Friedman’s test χ^2^_F_(3) = 1.49, *p* = 0.68). Also the ratio of probability of choosing A over avoiding B was not affected by the type of stimulation (Friedman’s test χ^2^_F_(3) = 0.85, *p* = 0.84).

**FIGURE 3:**
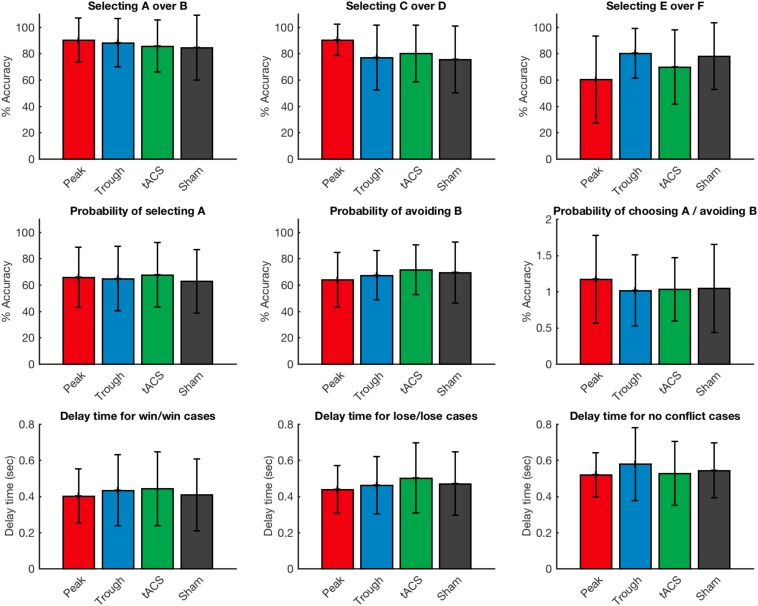
Accuracy and response timing of the volunteers during the test period of the game during the various possible choice scenarios on the task. Error bars represent standard deviation.

In addition, we evaluated the reaction times in no conflict cases where there was winning character paired with a losing character (i.e., AB), lose/lose conflict cases where there was a losing character paired with another losing character (i.e., BD) and win/win conflict cases where a winning character was paired with another winning character (i.e., AC). There was no significant effect of stimulation type on reaction time for no conflict cases (Friedman’s test χ^2^_F_(3) = 3.03, *p* = 0.39), lose/lose conflict cases (Friedman’s test χ^2^_F_(3) = 0.52, *p* = 0.92), or win/win conflict cases (Friedman’s test χ^2^_F_(3) = 3.43, *p* = 0.33) (Figure 3).

### ERP Recording

**FIGURE 4:**
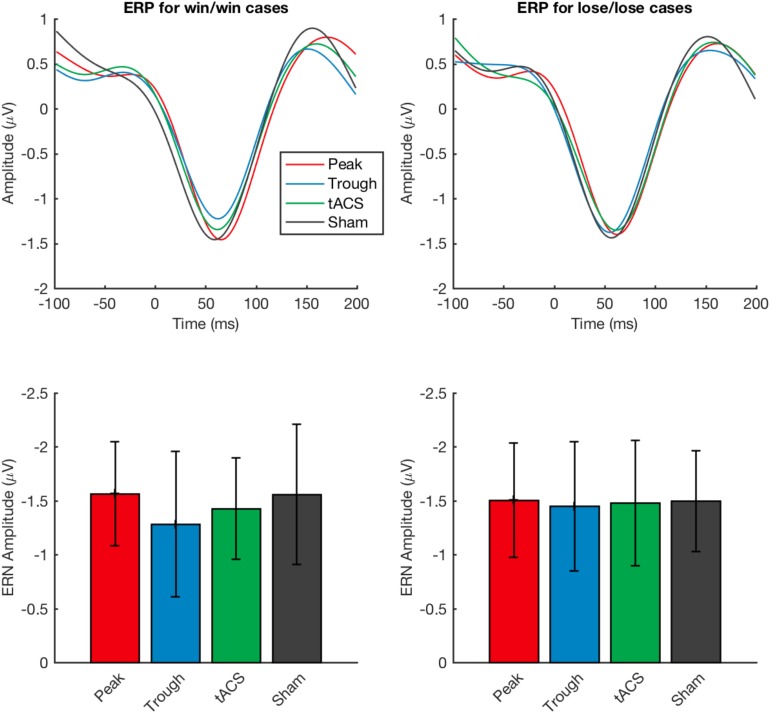
Event-related potential recordings obtained during the test task under the four types of stimulation applied. Waveforms for event-related positive (ERP) and amplitudes for event-related negative (ERN) potentials are shown for tACS applied under the sham, active non-phase-locked, active peak-phase-locked, and active trough phase-locked stimulation conditions are compared. No significant differences were detected across any of the four stimulation types. Error bars represent standard deviation.

Next, similarly to Frank et al., 2004, we investigated the event-related potentials when time-locked to response (Frank et al., 2004). The analysis showed similar ERPs as previously reported (Frank et al., 2004, 2005). There was no effect of stimulation in negativity amplitude in win/win ERP voltages (Friedman’s test χ^2^_F_(3) = 1.03, *p* = 0.79) and negativity amplitude in lose/lose ERP voltages (Friedman’s test χ^2^_F_(3) = 0.34, *p* = 0.95) (Figure 4).

### EEG Spectral Power

**FIGURE 5:**
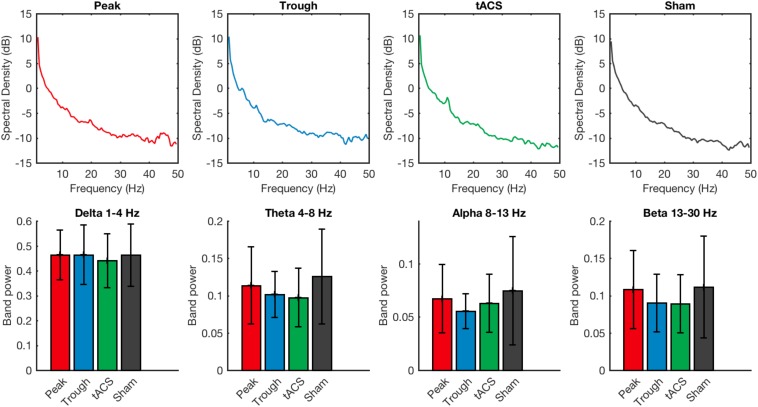
Power spectral densities of the resting EEG recorded after the training session are compared for tACS applied under the sham, active non-phase-locked, active peak-phase-locked, and active trough phase-locked stimulation conditions. No significant differences were detected across any of the four stimulation types. Error bars represent standard deviation.

Power spectral analysis of the resting EEG signal also did not show any difference among the 4 stimulation types. There was no statistical significance when measuring the power in delta (1–4 Hz) (Friedman’s test χ^2^_F_(3) = 1.12, *p* = 0.77), theta (4–8 Hz) (Friedman’s test χ^2^_F_(3) = 4.54, *p* = 0.21), alpha (8–13 Hz) (Friedman’s test χ^2^_F_(3) = 1.78, *p* = 0.62), or beta (13–30 Hz) (Friedman’s test χ^2^_F_(3) = 1.71, *p* = 0.64) EEG frequency bands (Figure 5).

## Discussion

In this work we have attempted to modulate SN activity differentially, by applying theta tACS or phase-locked tPCS synchronized to the frontal theta when the volunteers were engaged in a SN activating task. However, the results of the present study did not demonstrate differential effects of any of the 4 stimulation types on either electrophysiological or behavioral measures. Indeed, no significant effects were observed for any of the active stimulation conditions compared to sham stimulation in this investigation.

Previously, theta tACS applied during rest has been shown to be an effective modulator of frontal theta power (Pahor and Jaušovec, 2014); however, its effects have not been studied when the stimulation is applied during a task. In another study, theta tACS applied over parietal brain regions before a working memory task resulted in increased working memory storage; however, the same stimulation over frontal region had no effect (Jaušovec and Jaušovec, 2014). Cognitive effects of theta tPCS have been shown to be very small and specific to complex mathematical tasks (Morales-Quezada et al., 2015). In a more recent study, theta tACS applied over frontal region showed a decrease in nodal efficiency of dorsal anterior cingulate cortex (dACC) – one of the nodes of SN (Onoda et al., 2017).

The lack of any effect for any of the active stimulation conditions in the present study raises the question of whether the stimulation parameters were adequate to achieve neurophysiological effects in general, notwithstanding the role of phase-locking. Recent publications suggest that stronger transcranial electrical stimulation currents (i.e., >2 mA) may be required in some circumstances to have detectable effects on brain activity and behavior (Vöröslakos et al., 2018). In our study we respected the stimulation limits imposed by the hardware and what has been conventionally used and considered safe for these types of stimulation, which is 2 mA of current. However, it has been shown that in order to produce effective fields in the brain, currents as high as 6 mA are sometimes needed (Vöröslakos et al., 2018). Considering the thickness of the skull and the layers of dura protecting the brain, it is reasonable that stimulation may require higher currents to achieve the desired effect. Previously, other studies have shown that stimulation intensities as low as 2 mA can achieve sufficiently strong fields in the brain through computer simulations (Wagner et al., 2007), and studies using tDCS and tACS have been proven effective (Zaghi et al., 2010; Stagg and Nitsche, 2011; Reato et al., 2013; Kuo et al., 2014); however, these experiments have used different electrode montages and targeted other brain networks rather than SN itself. Moreover, brain stimulation in these studies was generally applied during rest, while in our study the stimulation was applied during a task. Further, some studies could only detect improvement in MDD patients after multiple sessions of tDCS (Brunoni et al., 2011; Alonzo et al., 2012), multiple sessions of stimulations should be considered for future trials. Finally, and importantly, most previous work regarding effective current amplitude applied either ongoing transcranial direct current stimulation (tDCS) or else sinusoidal tACS. In contrast, the present study’s phase-locked stimulation applied short pulses of 5 ms rather than sinusoidal pulses – a feature that may have precluded measurable effects on brain activity due to the short duty cycle of stimulation.

Considering the small sample size and the variability in the outcome measure, we speculate that the methods we used here (specifically, the 2 mA amplitude and brief-pulse waveform of phase-locked stimulation) may have had smaller effects than anticipated, and that the natural variability between the experimental sessions could have been larger than the effects of stimulation. In future work, using a larger sample size, increasing the stimulation intensity, or applying a longer duty cycle for the phase-locked tPCS could possibly lead to unveiling the effect of these types of NIBS.

As an additional factor to consider, the stimulation techniques used in this work may not have been suitable to modulate SN activity due to their low power and low spatial focality. Even though there is evidence of effectiveness of tACS in other models, modulation of SN has been mainly shown using rTMS, which is a much stronger type of brain stimulation in terms of both field intensity and depth. Body tissues have nearly uniform magnetic permeability and do not significantly distort the magnetic field produced by rTMS, allowing effective focusing of the stimulation to a restricted target at higher intensity than tES (Wagner et al., 2004). On the other hand, for non-invasive electrical stimulation, the large differences in electrical conductivity of the various tissues in the overlying scalp and skull not only blocks most of the current from reaching brain tissue, but also disperses the fields, thereby precluding effective focusing of the stimulation. Some work has been done to use multiple electrodes in a high density (HD) fashion to improve the focality of the stimulation (Kuo et al., 2013), which potentially could improve the effectiveness of the stimulation in future work.

It is believed that the electrical stimuli of tES affect the ongoing activity of the brain; thus, task-specific activation of the SN was used as means to assess the effect of the stimulation. However, the task used in our paper has not yet been used in the context of brain stimulation, and thus, perhaps other tasks should be studied with a similar stimulation protocol. While we know that the presently employed task activates SN and produces increased theta oscillation – a hallmark of SN activity – it is possible that in this case the task did not activate the specific nodes of the SN where the stimulation was delivered, or that the activation was not reflected in the outcome measures we studied in this experiment. We have selected these specific outcome measures based on the previous findings (resting EEG power changes, ERPs specific to the task and task specific behavioral changes). The effects of these types of stimulation has previously been shown to result in spectral power density changes in specific frequency bands during rest EEG (Zaehle et al., 2010; Helfrich et al., 2014; Vossen et al., 2015), changes in task specific activation of the brain (Meinzer et al., 2012; Jaušovec and Jaušovec, 2014; Cabral-Calderin et al., 2016), and behavioral or task specific performance changes (Santarnecchi et al., 2013; Voss et al., 2014). It therefore would be valuable to test our methods, tACS and phase-locked tPCS, in a sensorimotor model so we can check for changes in motor or sensory evoked potentials as a result of the stimulation.

Translationally, the search for novel NIBS approaches is much needed, as the current treatment options for TRD are very limited. Electroconvulsive therapy (ECT) has conventionally been the treatment of choice for TRD; however, it is associated with high costs, significant stigma, and cognitive side effects that deter many patients (Gangadhar, 2005). rTMS is a potential alternative; however, its limited availability, higher costs, and requirement for in-clinic rather than at-home application are the main limitations restricting its widespread adoption. A cost-effective, home-based brain stimulation technique may potentially resolve those limitations and provide an alternative treatment option for TRD patients. tPCS/tACS can potentially be made available as a home-based treatment, and the application of the stimulating currents in an oscillatory, phase-locked fashion could provide an opportunity to improve its efficacy by attempting to engage specific brain networks. Primarily, the past few decades of neuropsychiatric and neuroimaging research points toward the SN as the main target for treating MDD and TRD. However, tDCS and other simple electrical stimulation protocols can become more effective when integrated in a closed-loop system (Zrenner et al., 2016). Future work in this direction may potentially help develop a closed-loop electrical based brain stimulation technique.

To summarize, in this study we have tested novel approaches, tACS and phase-locked tPCS, to stimulate the SN network with hopes that such stimulation could be used for therapeutic purposes. If found effective, these techniques could potentially open doors for new treatments of MDD and other neuropsychiatric disorders linked to the SN. Although our results showed no statistically significant effect of stimulation on the ERP recordings, resting EEG recordings, and the performance of the volunteers in a SN activating task, future work will reveal whether such effects could be obtained by either modifying the amplitude or waveform of electrical stimulation, or else applying the same phase-locking algorithm to a more powerful stimulation modality, such as rTMS. Success could lead to more successful outcomes for patients undergoing treatment for TRD and other medically refractory neuropsychiatric illnesses.

## Data Availability Statement

The datasets generated for this study are available on request to the corresponding author.

## Ethics Statement

Twenty healthy volunteers free of neurological or psychiatric illnesses participated in the study. All participants gave informed consent, and the study was approved by the Research Ethics Board of the University Health Network.

## Author Contributions

All authors listed have made a substantial, direct, and intellectual contribution to the work, and approved it for publication.

## Conflict of Interest

The authors declare that the research was conducted in the absence of any commercial or financial relationships that could be construed as a potential conflict of interest.
